# Salvage therapy for progressive, treatment-refractory or recurrent pediatric medulloblastoma: a systematic review protocol

**DOI:** 10.1186/s13643-020-01307-8

**Published:** 2020-03-04

**Authors:** Ashley A. Adile, Michelle M. Kameda-Smith, David Bakhshinyan, Laura Banfield, Sabra K. Salim, Forough Farrokhyar, Adam J. Fleming

**Affiliations:** 1 McMaster Stem Cell and Cancer Research Institute, McMaster University, Hamilton, ON Canada; 2 Department of Biochemistry and Biomedical Sciences, McMaster University, Hamilton, ON Canada; 3 Department of Surgery, Division of Neurosurgery, McMaster University, Hamilton, ON Canada; 4 Health Sciences Library, McMaster University, Hamilton, Ontario Canada; 5 Department of Health, Evidence and Impact, McMaster University, Hamilton, Ontario Canada; 6 Department of Pediatrics, Division of Hematology-Oncology, McMaster University, Hamilton, Canada

**Keywords:** Salvage therapy, Medulloblastoma, Recurrent disease, Children

## Abstract

**Background:**

Central nervous system tumors remain the leading cause of cancer-related mortality amongst children with solid tumors, with medulloblastoma (MB) representing the most common pediatric brain malignancy. Despite best current therapies, patients with recurrent MB experience have an alarmingly high mortality rate and often have limited therapeutic options beyond inadequate chemotherapy or experimental clinical trials. Therefore, a systematic review of the literature regarding treatment strategies employed in recurrent pediatric MB will evaluate previous salvage therapies in order to guide future clinical trials. The aim of this systematic review will be to investigate the efficacy and safety of salvage therapies for the management of children with progressive, treatment-refractory, or recurrent MB.

**Methods:**

We will conduct literature searches (from 1995 onwards) in MEDLINE, EMBASE, ClinicalTrials.gov, WHO International Clinical Trials Registry Platform, and Cochrane Central Register of Controlled Trials. Studies examining the survival and toxicity of therapies administered to treatment-refractory pediatric MB patients will be included. Two reviewers will independently assess the search results based on predefined selection criteria, complete data abstraction, and quality assessment. The primary outcomes of this review will be overall and progression-free survival. Secondary outcomes will include safety and toxicity of each therapy administered. The study methodological quality (or bias) will be appraised using an appropriate tool. Due to the nature of the research question and published literature, we expect large inter-study heterogeneity and therefore will use random effects regression analysis to extract the combined effect. In additional analyses, we will investigate the role of re-irradiation and mono- vs. poly-therapy in recurrent disease, and whether molecular subgrouping of MB influences salvage therapy.

**Discussion:**

This systematic review will provide an overview of the current literature regarding salvage therapies for relapsed MB patients. Investigation of clinically tested therapies for children with recurrent MB has significant implications for clinical practice. By reviewing the efficacy and toxicity of MB salvage therapies, this study will identify effective therapeutic strategies administered to recurrent MB patients and can inform future clinical trials aimed to improve patient survivorship and quality of life.

**Systematic review registration:**

PROSPERO CRD42020167421

## Background

Central nervous system (CNS) tumors are the most common solid malignancies amongst children and remain the leading cause of pediatric cancer-related mortality in this group [1–3]. As the most common malignant brain cancer in children, medulloblastoma (MB) accounts for 20% of CNS tumors and occurs most commonly in the pediatric population, particularly in those younger than 15 years of age [4, 5]. Treatment involves maximal safe tumor resection, craniospinal irradiation, and high-dose chemotherapy. Although applying multi-modal therapies (radiation, chemotherapy, and surgery) has increased 5-year survival amongst standard-risk MB patients, overall survival (OS) rates seem to have plateaued. There has been minimal improvement in outcomes for high-risk patients, who often relapse and have high rates of disease metastasis [6]. Approximately 30–40% of MB patients experience relapse and have proven particularly difficult to treat [7]. These patients with treatment-refractory MB carry a high mortality rate, accounting for nearly 10% of all pediatric solid tumor-related deaths [7].

Within the past decade, basic science research has provided insight into the complexity of treatment-refractory MBs, demonstrating that recurrent MBs are often genetically distinct from their matched primary tumor [8, 9]. Therefore, any targeted treatment regimen identified to be effective on primary MB samples is poised to fail in recurrent lesions. Recent clinical trial design has aimed to integrate this emerging molecular genetic data into direct therapeutic targeting of the progressive disease [10]. However, treatment protocols for progressive MB currently lack standardization, making it difficult to determine the best course of treatment for these children. Current clinical trials employ salvage therapies, such as re-irradiation, re-administration of cytotoxic chemotherapy regimens, and new targeted treatments, which are aimed to elucidate the effectiveness and tolerance of each administered compound in children with recurrent disease [11–19]. To our knowledge, there is no published article that has systematically assessed the therapeutic options administered to pediatric patients with treatment-refractory MB [20]. Systematically reviewing the literature of previously employed salvage therapies and analyzing their efficacy and safety may contribute to our understanding of effective treatment regimens for recurrent pediatric MB and hold the potential to inform future clinical trials.

## Objective

This systematic review aims to assess the efficacy and safety of salvage therapies that have been used to manage children with progressive, treatment-refractory, or recurrent MB, as measured by overall and progression-free survival, as well as grading of treatment-related toxicities.

## Methods

Standard methodology of systematic reviews and meta-analyses will be conducted to ensure limited bias in study identification, selection, and extraction. The present protocol has been reported in accordance with the guidelines outlined in the Preferred Reporting Items for Systematic Reviews and Meta-Analysis Protocols (PRISMA-P; see checklist in Additional file 1) [21]. A summary of the protocol has been registered within the International Prospective Register of Systematic Reviews database (PROSPERO; registration number CRD42020167421).

### Eligibility criteria

Studies will be included based on the detailed PICOS (Population, Intervention, Comparison, Outcome, and Study design) framework:

#### Population

The study population includes infants, children, adolescents, and young adults (up to and including age 21 years to capture different definitions) with diagnoses of progressive, treatment-refractory, or recurrent MB at the time of study recruitment or following initial treatment [20]. To reflect the earlier classification of MB as an infratentorial small round blue cell tumor, articles on recurrent MB, posterior fossa tumors, primitive neuroectodermal tumors (PNETs), and CNS sarcomas will be included during title and abstract screening [22]. Studies that aggregate children and young adults or children with different types of solid or CNS tumors will be included, provided that participant data and outcomes can be evaluated separately for each group. In addition, articles that combine MB/PNET patients will be included during title and abstract screening to ensure inclusion of all relevant articles; if results are not reported separately for each tumor type in the full text, studies will be flagged and excluded. Full-text articles will also be excluded where MB patients are not included, or inclusion of these patients is not specified. Studies that incorporate both newly diagnosed and recurrent tumors, adult patient cases, or possess any ambiguity will be included during the title and abstract screen, flagged for discussion between reviewers (AAA, SKS, DB), and further evaluated during the full text review.

#### Intervention

Any therapeutic modalities administered as salvage therapies to the specified population will be included (i.e., second surgery, re-irradiation, re-administration of standard- vs. high-dose chemotherapy, immunotherapy, bone marrow/stem cell transplantation, targeted therapies, and other therapeutic modalities alone or as part of a multimodal approach) [11–19, 23–27]. All studies discussing socioeconomic factors of the disease or aspects unrelated to clinical outcomes (OS, progression-free survival (PFS), and/or associated toxicity) will be excluded during manual screening.

#### Comparison

With no study design filters in the search strategy, we aim to include randomized controlled trials (RCTs) in our systematic review; however, such trials have not often been conducted in this field yet. As the majority of eligible studies only include patients receiving salvage therapy or palliative care, no specific comparator will be defined a priori. Therefore, any comparator (i.e., active or inactive therapy), including no therapy as the control group, will be included.

#### Outcome

The primary outcome will be OS and PFS. Secondary outcomes will include toxicity and safety of each therapeutic regimen. The Common Terminology Criteria for Adverse Events (CTCAE) v5.0 guidelines, as defined by the National Cancer Institute of the National Institutes of Health (NCI-NIH), will provide a standard of reporting safety and toxicity data of administered treatments (i.e., procedural complications and administration site conditions; development of infections, such as respiratory disorders; emergence of benign, malignant, and unspecified (i.e., cysts and polyps) neoplasms; musculoskeletal and connective tissue disorders; metabolism and nutrition disorders; nervous system disorders; psychiatric disorders, including depression, addictive behaviors, and bipolar and related disorders) [28]. Additional outcome measures include evaluating the role of re-irradiation, mono- vs. poly-therapy, and whether molecular subgroups affect the efficacy of administered salvage therapies.

#### Study design

As no study design filters will be placed during the literature search, randomized and non-randomized controlled trials, prospective and retrospective studies, observational and interventional clinical trials, and case reports will be included. Studies lacking clinical outcome data, such as review articles, letters, editorials, and perspective and commentary pieces, will be excluded. During initial title and abstract screens, molecular or basic science publications, specific grey literature (i.e., letters to the editor, conference, and meeting abstracts), and clinical trials in recruitment phase or without results will also be excluded. Only completed studies with results from ClinicalTrials.gov, World Health Organization International Clinical Trials Registry Platform (WHO ICTRP), Cochrane Library, and bibliographic databases will be included and further assessed during full-text screening.

### Information sources, search strategy, and search terms

A broad, comprehensive literature search will be developed by an academic health sciences librarian with expertise in systematic reviews and search strategy design. Searches are expected to elucidate studies that evaluate the efficacy and safety of salvage therapies for children with progressive, treatment-refractory, or recurrent MB. The search within bibliographic databases will consist of indexed English articles available from 1995 onwards, without publication-type restrictions. Articles published prior to 1995 will be excluded, as only the following years represent the modern era for MB standard of care with surgery, adjuvant chemotherapy, and craniospinal radiation [29–33]. Studies published during or after 1995 that report on results dating before 1995 will be excluded, while articles with information from before and after 1995 will be included for further assessment in full-text review. A combination of keywords and medical subject heading terms will be used, relating to salvage therapies administered to pediatric patients (i.e., infants, children, young adults, and/or adolescents, whom are no older than 21 years) with progressive, treatment-refractory, or recurrent MB or PNET. Searches for appropriate studies will be conducted in the following databases: Medical Literature Analysis and Retrieval System (MEDLINE), the Excerpta Medica Databases (EMBASE), and Cochrane Central Register of Controlled Trials (CENTRAL) [34]. The draft search strategy for the MEDLINE database is available as Additional file 2.

To mitigate the risk of missing relevant studies, grey literature searches will be included in this review. Searches of the National Institutes of Health Clinical Trials (or ClinicalTrials.gov; http://www.clinicaltrials.gov) and WHO ICTRP (http://www.who.int/ictrp/en/) will identify completed and ongoing studies; however, only clinical trials with reported results will be assessed and included, if deemed relevant. We will identify eligible studies using the search strategy, “Recurrent medulloblastoma, childhood” and “PNET, Recurrent” for ClinicalTrials.gov, while WHO ICTRP searches included “recurrent medulloblastoma” and “PNET”. Synonyms automatically generated by WHO ICTRP are listed in Additional file 3.

### Data management

Results from the literature search of all databases will be exported as RIS or Microsoft Excel files. References from bibliographic databases will be uploaded to the online Covidence software [35]. The health sciences academic librarian (LB) will provide the review team with Excel files of grey literature searches, which will be manually screened thereafter. Duplicated articles will be automatically identified and eliminated by Covidence, or manually removed by reviewers (AAA, SKS, DB). Covidence is an online systematic review management software, which allows for improved efficacy, accessibility, and reproducibility for reviews. It will be used during title and abstract, as well as full-text review screening, while Microsoft Excel will be utilized for data extraction.

### Selection and data collection process

Two reviewers (AAA, SKS) will independently assess the search results, conduct data abstraction, and complete quality assessment, as guided by the population, intervention, and study design eligibility criteria. Reviewers will include all studies that appear to meet the inclusion criteria, or where further investigation is required to determine eligibility. Full reports of eligible studies will be retrieved and uploaded to Covidence by one reviewer (AAA) and confirmed by another (SKS), following article title and abstract screening. Information from full-text articles will be extracted independently and in duplicate by two reviewers (AAA, SKS).

Reviewers will develop a comprehensive data extraction form on Microsoft Excel, as guided by Covidence’s piloting forms, and data collection forms for RCTs by Cochrane Training, in addition to several peer-reviewed systematic reviews and protocols [36–41]. An initial calibration test will be conducted (AAA, SKS) on a set of 5 randomly selected articles and 5 randomly selected clinical trials to ensure high inter-rater agreement; the latter will compare manual and automatic data extraction methods (using Extracting Accurate efficacy and safety information from ClinicalTrials.gov, EXACT) [42]. A third reviewer (DB) will oversee both pilot tests. Collectively, the review team (AAA, SKS, DB, MMK-S, AJF) will refine the full-text screening criteria and report reasons for study exclusion. Modification of the developed data extraction forms will allow for a standardized piloting methodology, as guided by the results and subsequent discussion following the calibration tests—after which formal screening will commence. Based on study design, outcome measures, and data presented, we anticipate that data extraction and updating piloting forms will be an iterative process. Throughout the extraction process, reviewers (AAA, SKS) will meet and compare findings to ensure reliability and reproducibility amongst data collection approaches. Disagreements or discrepancies between reviewers, during any phase of the review process, will be recorded and a third reviewer will be an arbiter, if necessary. Furthermore, first and corresponding authors of the original articles will be contacted by one reviewer (AAA; with a maximum of three emails per author) if studies possess ambiguity for information pertinent to judge outcome results. Where additional data is required but unavailable, the review team will discuss the impact of missing data. For example, if the number of studies deemed to be eligible after full-text review is below the minimum required for statistical analyses and no response is received from the original authors, the review team will discuss and determine whether previously flagged articles featuring aggregated MB/PNET data should be extracted.

### Data items

A standardized data extraction form will be used to collect relevant information from each study, which will include (but is not limited to) the following: study characteristics (i.e., citation, author details, participating center(s)), methodology (i.e., study design, randomization method, data extraction methods, quality assessment results), population (i.e., eligibility criteria, age, male/female proportion, prior treatment history), intervention (i.e., therapeutic(s) label, dosage, duration and frequency, delivery method), and outcome measures (i.e., definition, type and number of events/type, toxicity/safety effects), as listed in Additional file 4. Furthermore, data from articles with healthy comparison cohorts will be abstracted, using the same criteria with the exception of tumor- and treatment-related variables.

### Outcomes and prioritization

The outcomes of the present study were selected based on our team’s expert knowledge of MB and in consultation with clinical neuro-oncology experts. Specifically, the primary outcomes of this review are OS and PFS of progressive, treatment-refractory, or recurrent pediatric MB patients from treatment-related mortality, defined as death associated with administered salvage therapy. Secondary outcomes involve treatment-related morbidities as specified by safety and toxicity (i.e., physical and/or cognitive, short- and/or long-term, serious or other adverse effects). Priority will be given to these outcome measures, as they hold the most clinical relevance and the majority of eligible studies are likely to include survival and/or toxicity data. OS and PFS will be reported as percentages in a given timeframe (i.e., 3, 5, or 10 years), whereas toxicity data will be transcribed using pre-defined definitions and numerical reports of co-morbidities (i.e., number of events per adverse effect). Specifically, reviewers will cross-reference data presented in each applicable study and the CTCAE v5.0 guidelines, as to provide standardized definitions of common adverse effects, when reporting secondary outcomes. The NCI-NIH’s guidelines outline the extent of organ toxicity experienced by cancer patients undergoing therapy, while providing clinical definitions of the severity for each adverse effect [28]. Some possible safety concerns that may arise include (but is not limited to) blood and lymphatic system disorders; cardiac disorders; ear and labyrinth disorders; endocrine disorders; eye disorders; gastrointestinal disorders; general disorders and administration site conditions; hepatobiliary disorders; immune system disorders; infections and infestations; injury; poisoning and procedural complications; metabolism and nutrition disorders; musculoskeletal and connective tissue disorders; neoplasms benign; malignant and unspecified (including cysts and polyps); nervous system disorders; psychiatric disorders; renal and urinary disorders; reproductive system and breast disorders; respiratory, thoracic, and mediastinal disorders; skin and subcutaneous tissue disorders; and vascular disorders [28]. Furthermore, CTCAE v5.0 assigns grades 1 through 5 with explicit clinical definitions of the severity for each adverse effect (i.e., grade 1: mild, asymptomatic or mild symptoms, clinical or diagnostic observations only, intervention not indicated; grade 2: moderate, minimal, local or non-invasive intervention indicated, limiting age-appropriate instrumental activities of daily living; grade 3: severe or medically significant but not immediately life-threatening, hospitalization or prolongation of hospitalization indicated, disabling, limiting self-care activities of daily living; grade 4: life-threatening consequences, urgent intervention indicated; grade 5: death related to adverse effects) [28]. As such, the preferred reporting of toxicity data will involve using CTCAE v5.0 definitions, where applicable. In the event that any article features definitions of treatment-related morbidities absent from the CTCAE v5.0 guidelines, adverse effects will be quoted verbatim.

Other outcomes include elucidating the role for re-irradiation and mono- vs. poly- therapy in recurrent disease, as such approaches are often employed in salvage therapies, in addition to discerning whether molecular subgrouping affects the effectiveness of the administered treatment regimen(s) [11–13, 26, 27]. We expect that these measures will require further investigation into collected data, granted that such data is available. As a result, priority will not be given to these outcomes. We anticipate data relevant to these outcomes will be heterogenous and will require cautious interpretation with subsequent description in the meta-analysis within the discussion section.

### Risk of bias assessment

The quality of randomized and non-randomized studies will be assessed by the Cochrane Risk of Bias RoB 2.0 tool and ROBINS-I tool, respectively [43, 44]. This approach provides highly detailed assessment criteria, which limits any subjectivity present and thereby will strengthen the quality of this review. Parameters of bias in each study will be scored as having low, medium, high, or unclear risk, in which the former is deemed as a high-quality study. While we will not exclude low-quality/high-risk studies, we will use these results as a means to describe the rigor of included articles. An online risk of bias visualization application by Cochrane, termed robvis, will be explored by reviewers with the intention to generate a figure that summarizes quality assessments, which may be included in the meta-analysis [45]. If this figure is not favored to be included in the systematic review, a tabular presentation of the risk of bias assessment for individual studies will be included. All methodological criteria will be at the overall study, rather than outcome level, as to not clutter the risk of bias assessment figure or table with each fully reported outcome. Additionally, quality and level of evidence will be described via the GRADE (Grading of Recommendations, Assessment, Development and Evaluations) recommendation criteria [46]. According to GRADE, it is expected that data from RCTs will produce high-quality evidence although quality can be diminished due to the robustness of the included studies. In order to determine the quality, strength, and therefore recommendation of eligible studies, key factors such as inconsistency between studies, publication bias, and imprecision of results will require in-depth investigation. The quality of evidence will be stratified into one of four categories: high, moderate, low, and very low (i.e., *high quality*: confidence in the estimate of intervention effect is not likely to waiver, even with additional research; *moderate quality*: further research is likely to hold an important role on the confidence of the effect’s estimate and may change it; *low quality*: further research is very likely to hold an important role on the confidence of the effect’s estimate and is likely to change it; *very low quality*: uncertainty exists from any estimate of intervention effect) [46]. The GRADE-CERQual will be used to inform the confidence of results [47, 48]. Furthermore, a funnel plot will be used for visual assessment of the publication bias and selective reporting.

Two reviewers (AAA, SKS) will independently complete the quality assessment. Methods similar to those undertaken in data piloting will be employed (i.e., calibration test using appropriate Cochrane tools and GRADE criteria, if necessary; regular meetings to compare findings). Discrepancies will be discussed and resolved by consultation with a third reviewer (DB).

### Data synthesis

The baseline participant characteristics (i.e., number of participants, median age, age range, male/female proportion, prior treatment history, year of publication, etc., for all participants and specifically MB participants) will be summarized in tables. Clinical heterogeneity (i.e., population, interventions, and outcome measurement features) and methodological heterogeneity (risk of bias) will be described and considered in data synthesis. Data on the outcome measures will be pooled, and the odds ratios of OS and PFS, with their corresponding 95% confidence intervals, will be reported for each intervention and compared with one another.

The odds ratios between the interventional groups will be estimated using random effects model with an inverse variance approach as we anticipate large between-study heterogeneity. Heterogeneity will be tested using the Cochrane’s *Q* test with *p* value set at 0.1 for significance and quantified using *I*^2^ statistic (*I*^2^ < 25% as low, 40–50% as moderate, and > 50% as substantial heterogeneity). *I*^2^ outputs will be analyzed, and the feasibility of combining clinical studies will be assessed. Inter-study outputs with high heterogeneity will be described independently. The synthesis of the secondary outcome measures will be conducted using similar approaches and methodologies. Stata SE 12.0 (StataCorp LP, College Station, Texas, USA) and Review Manager (RevMan) 5.3 (The Nordic Cochrane Center, Copenhagen, Denmark) will be used for data synthesis.

To examine the contribution of moderator variables on the absolute PFS difference, a random effects meta-regression model will be applied using “metareg” macro available for Stata SE 12.0 software. The dependent variable will be the PFS, and the moderator variables will include study location; baseline mean demographics, such as median age and its corresponding stable disease; male/female proportion; therapeutic interventions; and the Cochrane quality components and sample size. First, a univariable random effects meta-regression will be performed, and then, variables with a significance level of 0.1 will be tested in a multivariable meta-regression analysis. Between moderators, interaction will be considered for adjustment and included in the multivariable meta-regression analysis.

Sensitivity analysis will be performed by excluding trials with > 15% of the missing outcome data and in case of large heterogeneity. A *p* value of 0.05 will be used for statistical significance. Meta-biases, such as publication bias and selective reporting, will be evaluated by a funnel plot for visual assessment.

Where quantitative synthesis is not appropriate (i.e., when aiming to identify the role for re-irradiation and mono- vs. poly- therapy in recurrent disease, or whether molecular subgrouping influences the efficacy of salvage therapies), careful evaluation of the results with subsequent description will be included.

### Amendments

Any amendments to the protocol will be dated, and detailed rationale for which these changes are made will be documented in PROSPERO. Such changes will only be made following extensive group discussion (AAA, SKS, DB, MMKS, AJF). In the event that any necessary amendments affect statistical data analyses, our biostatistics expert (FF) will be consulted to determine a more suitable alternative. However, given that a standardized data extraction form will be developed by reviewers following full-text screening, modifications to the piloting form template (criteria listed in Additional file 4) will not be considered amendments. Therefore, the finalized extraction form will be included in the meta-analysis.

## Discussion

The present study aims to outline the methodology and analytical (both statistical and narrative) approaches that will be employed to detail a systematic review of salvage therapies for recurrent pediatric MB patients. This protocol is strengthened by utilizing the PRISMA-P guidelines, which improve both study accuracy and transparency. Additional strengths of this study stem from employing two reviewers to independently conduct screens, data extraction, and quality assessment (or risk of bias). To mitigate against subjectivity and human error, regular correspondence amongst reviewers throughout data collection will corroborate accuracy of findings. Inclusion of various experts in the fields of systematic reviews and search strategy design, MB, clinical research, and biostatistics has also served to improve this review. Every attempt was made to develop a comprehensive protocol with appropriate literature searches, screening, data extraction, and reporting methodologies, along with detailed eligibility criteria. Review-level limitations of the present study include literature search restrictions by inclusion of indexed English articles published during and after 1995; however, articles during this time period will capture the relevant, modern era of MB standard therapy. Additional selection bias involves exclusion of ongoing and incomplete grey literature (i.e., conference abstracts, reviews, incomplete clinical trials), but the data disclosed in these studies are likely insufficient to extract relevant study and outcome results (i.e., all participating centers, participant characteristics, prior treatment history, intervention information for each participant, aggregated vs. non-aggregated data, etc.). Therefore, exclusion of these studies is expected to improve the overall quality of the review. Furthermore, the absence of RCTs conducted in recurrent MB disease represents a publication bias. Given that treatment-refractory, pediatric MB is a highly specialized field, included studies will describe the limited number of centers proficient at treatment administration for pediatric MB patients. Lastly, heterogeneity both within and between studies (i.e., inclusion and exclusion criteria, participant characteristics like age at relapse(s), small sample size of MB patients in each study, reporting of MB tumors separately or aggregated with other tumors) may also limit generalization of results and thereby will undergo careful evaluation. Despite the potential caveats, it is still important to undertake this systematic review, given the impact that such work might have for future treatment of children with recurrent MB.

Though multi-modal therapy protocols have significantly improved the overall prognosis for MB, many patients still experience relapse of disease. Patients presenting with disseminated disease at time of recurrence are often provided with palliative care alone. These children who are identified as having relapsed on radiological and clinical follow-up have limited therapeutic options beyond the setting of clinical trials, and no consensus to standard of care exists. The aim of this review is intended to evaluate the efficacy and safety of therapeutic strategies to salvage patients with progressive, treatment-refractory, or recurrent pediatric MB. As a result, identifying the association between clinical outcome and treatment-related morbidities of administered therapeutic agent(s) holds great potential to guide future clinical trials.

## Supplementary information

**Additional file 1.** PRISMA-P 2015 Checklist.

**Additional file 2.** Search strategy for Ovid MEDLINE.

**Additional file 3.** Search strategy for WHO ICTRP.

**Additional file 4.** Data extraction form template.

## Data Availability

Not applicable.

## Abbreviations

CENTRAL: Cochrane Central Register of Controlled Trials
CNS: Central nervous system
CTCAE: Common Terminology Criteria for Adverse Events
EMBASE: Excerpta Medica Databases
EXACT: Extracting Accurate efficacy and safety information from ClinicalTrials.gov
GRADE: Grading of Recommendations Assessment, Development and Evaluation
MB: Medulloblastoma
MEDLINE: Medical Literature Analysis and Retrieval System
NCI-NIH: National Cancer Institute of the National Institutes of Health
OS: Overall survival
PFS: Progression-free survival
PNET: Primitive neuroectodermal tumor
PRISMA: Preferred Reporting Items for Systematic Reviews and Meta-Analyses
PRISMA-P: Preferred Reporting Items for Systematic reviews and Meta-Analysis Protocols
PROSPERO: International Prospective Register of Systematic Reviews
RoB 2.0 tool: Revised tool for Risk of Bias in randomized trials
ROBINS-I tool: Risk Of Bias in Non-randomized Studies-of Interventions
WHO ICTRP: World Health Organization International Clinical Trials Registry Platform
