# Supplementary material for: Salvage therapy for progressive, treatment-refractory or recurrent pediatric medulloblastoma: a systematic review protocol
Source: Syst Rev. 2020 Mar 4;9:47. doi: 10.1186/s13643-020-01307-8 (PMC7055028; doi:10.1186/s13643-020-01307-8)
Supplement: Supplementary file 2 — Additional file 2. Search strategy for Ovid MEDLINE. [file 13643_2020_1307_MOESM2_ESM.pdf]

Additional file 2 – Results from pilot database search. Performed January 16, 2019 in MEDLINE.

#	Searches
1	Medulloblastoma/ (6548)
2	medullomyoblastoma*.mp. (71)
3	medulloblastoma*.mp. (9318)
4	Neuroectodermal Tumors, Primitive/ (1691)
5	PNET*.mp. (2820)
6	((primitive neuroectodermal or primitive neuro ectodermal or primitive neuroepothelial) adj2 (tumo?r* or neoplasm*)).mp. (3336)
7	ependymoblastoma*.mp. (291)
8	medulloepithelioma*.mp. (348)
9	spongioblastoma*.mp. (159)
10	or/1-9 (13887)
11	progressive.mp. (262184)
12	Neoplasm Recurrence, Local/ (107559)
13	recurr*.mp. (645671)
14	treatment refractory.mp. (2166)
15	refractory.mp. (116031)
16	regenerat*.mp. (198949)
17	relaps*.mp. (167417)
18	recidive.mp. (1457)
19	or/11-18 (1277922)
20	10 and 19 (2409)
21	Pediatrics/ (50515)
22	p?ediatric*.mp. (359378)

23	exp CHILD/ (1805860)
24	child*.mp. (2289694)
25	exp infant/ (1084364)
26	infant*.mp. (1200963)
27	newborn*.mp. (723478)
28	neonat*.mp. (271576)
29	exp ADOLESCENT/ (1907293)
30	adolescen*.mp. (1974307)
31	teenage*.mp. (19977)
32	youth*.mp. (71291)
33	Young Adult/ (712595)
34	young adult*.mp. (773866)
35	or/21-34 (4362511)
36	20 and 35 (1772)
37	limit 36 to (english language and yr="1995 -Current") (1258)
38	remove duplicates from 37 (1257)
