# Supplementary material for: Salvage therapy for progressive, treatment-refractory or recurrent pediatric medulloblastoma: a systematic review protocol
Source: Syst Rev. 2020 Mar 4;9:47. doi: 10.1186/s13643-020-01307-8 (PMC7055028; doi:10.1186/s13643-020-01307-8)
Supplement: Supplementary file 3 — Additional file 3. Search strategy for WHO ICTRP. [file 13643_2020_1307_MOESM3_ESM.pdf]

Additional file 3 – WHO ICTRP Search Strategy.

Table 1. Synonyms automatically searched by WHO ICTRP for "recurrent medulloblastoma"

MEDULLOBLASTOMA RECURRENT
recurrent medulloblastoma

Table 2. Synonyms automatically searched by WHO ICTRP for "PNET"

PRIMIT NEUROECTODERMAL TUMR
EWING SARCOMA / PERIPHERAL NEUROECTODERMAL TUMOR
EWING SARCOMA / PERIPHERAL NEUROECTODERMAL TUMOR (MORPHOLOGIC ABNORMALITY)
EWING SARCOMA / PERIPHERAL NEUROECTODERMAL TUMOUR
EWING TUMOR OF BONE
EXTRACRANIAL PRIMITIVE NEUROECTODERMAL TUMOR
NEOPL PRIMITIVE NEUROEPITHELIAL
NEOPLASM, PRIMITIVE NEUROEPITHELIAL
NEOPLASMS, PRIMITIVE NEUROEPITHELIAL
NEUROECTODERMAL NEOPL PERIPHERAL PRIMITIVE
NEUROECTODERMAL NEOPLASM PERIPHERAL PRIMITIVE
NEUROECTODERMAL TUMOR
NEUROECTODERMAL TUMOR, PERIPHERAL
NEUROECTODERMAL TUMOR, PERIPHERAL PRIMITIVE
NEUROECTODERMAL TUMOR, PRIMITIVE, NEUROECTODERMAL TUMORS
PERIPHERAL, NEUROECTODERMAL TUMORS
PRIMITIVE, NEUROECTODERMAL TUMORS
PRIMITIVE [DISEASE/FINDING], NEUROECTODERMAL TUMORS

PRIMITIVE, PERIPHERAL, NEUROECTODERMAL TUMORS

PRIMITIVE, PERIPHERAL [DISEASE/FINDING] NEUROECTODERMAL

TUMOR, PRIMITIVE, UNSPECIFIED SITE

NEUROEPITHELIAL NEOPL PRIMITIVE

NEUROEPITHELIAL NEOPLASM

PRIMITIVE, NEUROEPITHELIAL NEOPLASMS

PRIMITIVE, NEUROEPITHELIAL TUMOR

PRIMITIVE, NEUROEPITHELIAL TUMORS

PRIMITIVE, NEUROEPITHELIOMA

NEUROEPITHELIOMA (DIAGNOSIS)

NEUROEPITHELIOMA (MORPHOLOGIC ABNORMALITY)

NEUROEPITHELIOMA NOS

NEUROEPITHELIOMA, NOS

PERIPHERAL NEUROECTODERMAL TUM

PERIPHERAL NEUROECTODERMAL TUMOR

PERIPHERAL NEUROECTODERMAL TUMOR (DIAGNOSIS)

PERIPHERAL NEUROECTODERMAL TUMOR (DISORDER)

PERIPHERAL NEUROECTODERMAL TUMOR (MORPHOLOGIC ABNORMALITY)

PERIPHERAL NEUROECTODERMAL TUMORS

PERIPHERAL NEUROECTODERMAL TUMOUR

PERIPHERAL PRIMITIVE NEUROECTODERMAL NEOPL

PERIPHERAL PRIMITIVE NEUROECTODERMAL NEOPLASM

PERIPHERAL PRIMITIVE NEUROECTODERMAL TUMOR

PERIPHERAL PRIMITIVE NEUROECTODERMAL TUMORS

PERIPHERAL PRIMITIVE NEUROECTODERMAL TUMOUR

PRIMIT NEUROECTODERMAL TUMR

PRIMITIVE NEUROECTODERMAL TUM

PRIMITIVE NEUROECTODERMAL TUMOR

PRIMITIVE NEUROECTODERMAL TUMOR (DISORDER)

PRIMITIVE NEUROECTODERMAL TUMOR (MORPHOLOGIC ABNORMALITY)

PRIMITIVE NEUROECTODERMAL TUMOR, EXTRACRANIAL

PRIMITIVE NEUROECTODERMAL TUMORS

pnet
