# Supplementary material for: Salvage therapy for progressive, treatment-refractory or recurrent pediatric medulloblastoma: a systematic review protocol
Source: Syst Rev. 2020 Mar 4;9:47. doi: 10.1186/s13643-020-01307-8 (PMC7055028; doi:10.1186/s13643-020-01307-8)
Supplement: Supplementary file 4 — Additional file 4. Data extraction form template. [file 13643_2020_1307_MOESM4_ESM.pdf]

Additional file 4 – Template of study identifiers and data to be extracted.

Criteria	Determinants
Study characteristics	Reference (i.e., Vancouver style or clinical trial identifier)
	Year of publication
	Sponsorship/funding source(s) (if reported)
	Author details (i.e., first and corresponding authors' names, email(s), institution, address)
	Participating centers (i.e., unicenter vs. multicenter, list included centers and countries)
	Year(s) of study duration
Methods	Study design (i.e., randomized and non- randomized controlled studies, prospective and retrospective cohort studies, case reports, cross-sectional studies, case-control studies, cohort/longitudinal studies, etc.)
	Randomization method (if applicable, state parallel, factorial, crossover, cluster, etc.)
	Statistical design (i.e., 3+3, continual reassessment method, Bayesian methods, interval-based designs)
	Data extraction methods
	Results of quality assessment
Population	Patient inclusion and exclusion criteria
	Target population tumour categories (i.e., MB, PNET, CNS tumours, solid tumours)
	Target population tumour categories reporting method (i.e., CNS tumours reported as aggregates, MB and PNET reported as aggregates or separately, etc.)
	Total sample size
	MB participant sample size (report as number and percentage of total sample size)
	Male/female proportion

	Median age of target population and range of ages
	Age at inclusion (i.e., up to 18 years, up to 21 years, etc.)
	Age at relapse (if reported)
	Prior treatment history
	MB molecular subgroup (if reported)
	Histology (if reported; classic, desmoplastic/extensive nodular, or large cell anaplastic)
	Metastatic stage (if reported)
Interventions	Description of administered interventions
	Dosage, duration and frequency
	Route(s) of administration (i.e., oral, nasal, sublingual, injection, inhalation, etc.)
Outcomes	Name/definition
	Type (i.e., continuous, dichotomous, adverse event, other, or combination)
	Number of events per outcome type
	OS and PFS (if reported; i.e., 3, 5, 10-year, etc.)
	Serious and other adverse effects of treatment (if reported)
	Occurrence of dropouts and/or missing data (if reported; state reasons)
Other	Key conclusion(s)
	Additional comments (i.e., reference to other studies, bias or conflict of interest, etc.)

MB, medulloblastoma; PNET, primitive neuroectodermal tumour; CNS, central nervous system.
